# Acquisition of Mathematical and Linguistic Skills in Children With Learning Difficulties

**DOI:** 10.3389/fpsyg.2021.793796

**Published:** 2021-12-28

**Authors:** Nurit Viesel-Nordmeyer, Ute Ritterfeld, Wilfried Bos

**Affiliations:** ^1^Department of Rehabilitation Science, TU Dortmund University, Dortmund, Germany; ^2^Lyon Neuroscience Research Center (CRNL), University of Lyon, Lyon, France; ^3^Center for Research on Education and School Development, TU Dortmund University, Dortmund, Germany

**Keywords:** learning difficulties, linguistic skills, math skills, working memory, comorbidity

## Abstract

Comorbid learning difficulties in linguistic and mathematical skills often emerge in primary school age. The cause of coinciding of both learning difficulties during children’s development spanning pre- and primary-school age is not yet well understood. To address this research gap, we used data from the German National Educational Panel Study (NEPS; *n* = 301) of four groups of children which were categorized according to their skill levels in pre-school age: children with learning difficulties isolated in linguistic skills (LD), children with learning difficulties isolated in mathematical skills (MD), children with learning difficulties combined in linguistic and mathematical skills (MD/LD), and children with typical development in both skills (TA). Computing univariate and repeated measures ANCOVAs we compared the mathematical and linguistic development of the four groups of children (LD, MD, LD/MD, and TA) spanning age four to ten. Results reveal a partial catch-up in linguistic skills (lexical, grammatical) for children with LD. In contrast, children with MD did not overcome their mathematical competence gap in comparison with TA and LD. Moreover, children with MD showed a decrease in grammatical skills during transition in primary school. Further, children with MD/LD displayed the weakest performance in linguistic and mathematical skills during pre- and primary-school age in general. However, after controlling for working memory, initial performance differences between the groups decreased in favor of MD/LD. The relation between linguistic skills and mathematical skills in persisting learning difficulties as well as the specific role of working memory are discussed.

## Introduction

Many primary-school children experience difficulties meeting linguistic and mathematical requirements even if they have not been diagnosed with an intellectual disability. Such low achievers are estimated to account for about 23% of all students in linguistic and 8% in mathematical skills (e.g., Fischbach et al., 2013). Prevalent combined difficulties (6%, ibid.) point to a strong relationship between the acquisition of mathematical and linguistic skills. Researchers also found this close association in children with specific language disorders (Durkin et al., 2013) and in children undergoing second language acquisition or from a lower socioeconomic background (Demir et al., 2015).

### Difficulties With Linguistic Skills

About 90% of children with a pre-school history of language difficulties achieve age-appropriate linguistic skills by school entry (e.g., Rescorla, 2002). Research shows that children whose language difficulties persisted throughout all their pre-school years have the highest risk of early language difficulties continuing into school age (Kühn, 2010; Snowling et al., 2016). There seems to be a critical age threshold at age four (Kühn, 2010) or five (Snowling et al., 2016) that differentiates between children who overcome their problems over time and those whose difficulties persist into school age. However, even some of the children who achieved adequate linguistic skills before school enrollment still deviate in their further linguistic development compared to their typically achieving peers (Rescorla, 2002; Kühn, 2010). Although there is evidence supporting a genetic risk for developmental language disorders, the socioeconomic background of the family holds additional predictive value (Dilnot et al., 2017). A lower socioeconomic background, which was already identified as responsible for input-dependent disadvantages in linguistic achievement during school age (e.g., Demir et al., 2015), particularly contributes to the risk for cumulative academic learning difficulties in both linguistic and mathematical skills (Aro et al., 2009).

### Difficulties With Mathematical Skills

Despite a great heterogeneity in mathematical learning difficulties (Kucian and von Aster, 2015) precursors can already be identified at pre-school age (e.g., Stock et al., 2010). A strong predictor for the evolution of mathematical learning difficulties is the specific capability for magnitude comparison. Other specific early skills which have indicated future challenges with learning mathematics include counting, seriation, and classification (Stock et al., 2010). Geary et al. (2012) addressed the persistence of learning difficulties between grades 1 to 5 by comparing the development of children with moderate vs. severe mathematical learning difficulties [clustered as mathematical low achievers (LA) vs. mathematical learning disabilities (MD)] to their typically achieving peers (TA). Taking a closer look at specific mathematical tasks, both low achieving groups (but especially the MD) are at first less proficient compared to their peers. However, further development displayed a heterogeneous pattern with variations in the kinds of difficulties. In number sets, there was a nearly parallel developmental process of all groups over time, just with the two low achieving groups continuously displaying lower scores than the TAs. In contrast, both low achieving groups were able to partially catch up with simple addition and completely with complex addition by grade 5. In grade 5, children with moderate learning difficulties could also close the gap in number line, while children with severe difficulties only partially caught up. Taken together, there is some opportunity for overcoming initial mathematical difficulties, depending on their severity. Further studies indicated that the persistence of lower mathematical difficulties is also related to socioeconomic background (e.g., Stock et al., 2010).

### Relationships Between Difficulties With Linguistic and Mathematical Skills

Several studies provide evidence for an association in the development of linguistic and mathematical skills, especially in children with learning disorders. For example, Durkin et al. (2013) found lower mathematical achievements in children between ages seven to eight with developmental language disorders in comparison to their typically achieving peers, especially if the children continued to decline in their language abilities. In a follow-up study, children with a history of pre-school language difficulties also showed persistent lower mathematical achievements in school compared to their peers, even if the initial linguistic difficulties had been resolved (Snowling et al., 2001). Moreover, a subtype comparison by Jordan and Hanich (2003) demonstrated different development patterns for mathematical as well as more advanced linguistic skills, depending on the specific learning difficulties spanning second to third grade: The study revealed persistent poor reading achievement for children with mathematical difficulties accompanied by a small improvement in reading for children with reading difficulties. This group with reading difficulties showed a fluctuating developmental pattern in mathematics while children with mathematical difficulties partly overcame their learning deficits (Jordan and Hanich, 2003). These findings contrast the assumption of a heterogeneous developmental pattern across different mathematical tasks between ages five and seven for children with domain-specific and combined learning difficulties (Jordan et al., 2015).

### Working Memory Capacity in Children With Learning Difficulties

In addition to weaknesses in linguistics and/or mathematical skills, children with learning difficulties often show low performance in their working memory system which is essential for information processing (e.g., Durkin et al., 2013; Peng and Fuchs, 2014; Peng et al., 2018). Based on the hierarchical model of Baddeley (2012), the working memory system can be divided into three components to which different information processing tasks are assigned: The *central executive* as the higher-level component is responsible for controlling and coordinating information processing in general. This also includes the coordination of their domain-specific supportive components *phonological loop* and the *visuo-spatial sketchpad*. The phonological loop briefly stores auditory or verbal information and maintains it during information processing. The visuo-spatial sketchpad performs the same tasks for visual and spatial information. In line with the proposed responsibilities of the three working memory components, studies have indicated specific limitations in working memory of children with different types of learning difficulties (cf. Kwok and Ansari, 2019): Low performance of the phonological loop as well as the central executive is associated with linguistic learning difficulties while weak performance of the central executive as well as the visuo-spatial sketch-pad where mainly found in children with mathematical learning difficulties. Moreover, the extent of deficits in working memory have been discussed as a third factor explaining the comorbidity of learning difficulties in linguistic and mathematical skills (Peters et al., 2018) as well as the severity of both (e.g., Brandenburg and Hasselhorn, 2019). In addition, an influence of the socioeconomic background of the parental home on children’s performance in working memory was found in recent research. To be specific, previous studies give evidence of negative effects of specific factors in the parental home on the development of children’s working memory. Limitations in the environmental language input (e.g., Engel et al., 2008), a higher level of stress (e.g., Hackman et al., 2014) as well as missing or limited home learning activities were identified as risk factors (e.g., Sarsour et al., 2010).

### The Present Study

The available studies reveal a rather heterogeneous picture about the combined development of linguistic and mathematical skills in children with learning difficulties. Moreover, these studies predominantly address primary-school age and only cover a short time period. As of yet, we have found no research that would help explain how the co-acquisition of both skills–linguistic and mathematical–unfolds throughout the entire course of pre- and primary-school age. Evidence about a longer developmental process, which includes the period of acquisition of basic skills during pre-school age as well as the critical time point of occurrence of learning difficulties during primary-school age, has so far only been shown for the development of either one or the other domain–linguistics or mathematics. Therefore, we decided to address this process in greater detail with groups of children with different types of learning difficulties. Furthermore, in studying the co-acquisition of linguistic and mathematical skills we can build on indications from previous work. These include the importance of working memory for specificity and severity of learning difficulties. Thereby we will observe changes in developmental differences between groups with different learning profiles while still considering this cognitive system of information processing and controlling for families’ socio-economic background. The following research questions were defined:

1) Does mathematical and linguistic development differ between subgroups of children with domain-specific, combined, or without any learning difficulties? We particularly aim to identify which group carries the highest risk for persistent difficulties.
2) Do developmental differences between subgroups of children with domain-specific, combined, and without any learning difficulties change after controlling for working memory capacity? With respect to recent research depicting limitations within different parts of working memory in children with specific learning difficulties (Durkin et al., 2013; Peng et al., 2018), we will specifically focus on the central-executive and phonological components (Baddeley, 1986).

## Materials and Methods

### Methods

Data (*n* = 301; female = 51.8%) derive from a longitudinal sample (group 3, starting cohort 2) from the German National Educational Panel Study (NEPS) (Blossfeld et al., 2011). To cover the age span of research interest, we used the available annual measurements from the second year of pre-school (age 4/5) until the fourth grade of primary school (age 9/10). Data of children with typical cognitive profiles (fluid intelligence within ±1.5 *SD*) were included (cf., Fletcher et al., 2001), data of children with physical impairments (e.g., hearing loss) were excluded. An imputation to maintain the initial sample size was rejected due to the lack of predictability of the missing information by third variables (Garson, 2015). Groups with pre-school measured learning difficulties in mathematics (MD; *n* = 26; mathematical skills, t1 < −1 *SD*; linguistic skills, t1 ≥ −1 *SD*), linguistics (LD; *n* = 23; linguistic skills, t1 < −1 *SD*; mathematical skills, t1 ≥ −1 *SD*) and both domains (MD/LD; *n* = 18; mathematical skills, t1 < −1 *SD*; linguistic skills, t1 < −1 *SD*) were compared next to their typical achieving peers (TA; *n* = 234; linguistic skills, t1 ≥ −1 *SD*; mathematical skills, t1 ≥ −1 *SD*). Grouping variables were children’s level of linguistic (vocabulary t1 and grammar t1 summed up equally) and mathematical skills (t1) at pre-school age. The cut-off criteria of 1 *SD* (percent range 16) was chosen in line with a large number of recent studies (e.g., Moll et al., 2014; Donker et al., 2016) which was integrated in a recent meta-analysis comparing groups of children with isolated, combined and without learning difficulties in the domains of language and mathematics (cf., Viesel-Nordmeyer et al., Submitted).

### Instruments

Competence tests were carried out in single (pre-school age) vs. group (primary-school age) designs *via* test leaders, set with annual interval in the institutions. Data were scaled using models of item response theory (IRT) and linked with anchor-item design. For more information regarding the panel data, see: https://www.neps-data.de/Data-Center/Data-and-Documentation/Starting-Cohort-Kindergarten. In the following section, the indicated internal consistency using Cronbach’s alpha (α) refers to the selected sample (*n* = 301).

Linguistic skills in NEPS were measured with listening comprehension tests in pre- and primary-school age. The vocabulary measurement bases on the German research version (Dunn and Dunn, 2007) of the “Peabody Picture Vocabulary Test” (PPVT) (Bishop, 1989), which was used in recent research to detect reading difficulties in time (e.g., Lewis, 1980). To specify, the 175 PPVT items established for the BIKS-3-10 study (see Ebert and Weinert, 2013) were shorten to 80 items for the NEPS data in general (c.f., Berendes et al., 2013). This yielded in underlying test batteries of 77 items in t1 (pre-school with 4–5 years; α = 0.91), 66 items in t2 (grade 1 with 6–7 years; α = 0.85) and 72 items in t3 (grade 3 with 8–9 years; α = 0.84). The measurement of grammar skills is based on the “Test for Reception of Grammar” (Bishop, 1989), which has already been used in previous studies in relation with comprehension difficulties in reading skills (e.g., Stothard and Hulme, 1992). Grammar skills were measured for one time each at pre-school (48 items in t1 with 4/5 years; α = 0.87) and primary-school age (40 items in t2 in first grade with 6–7 years, α = 0.82). Backed up by IRT-analyses, a short version could be used (TROG-D; Fox, 2006). These comprised of nearly all syntactic category groups of the original TROG-D (c.f., Berendes et al., 2013). For more information see Berendes et al. (2013).

Mathematical skills (t1: 5–6 years, α = 0.79; t2: first grade, α = 0.77; t3: second grade, α = 0.78; t4: fourth grade, α = 0.74) were collected using a self-constructed instrument of NEPS, based on the idea of mathematical literacy in PISA (OECD, 2013) and the curricular standards in STEM. Consequently, the measured competencies of the unidimensional construct represent subdomains like arithmetic, word problems, geometry, and quantity number concepts. For more information see Neumann et al. (2013).

Data available from one measurement time point for working memory (5/6 years) were included as covariates: The phonological loop was assessed using number recall from the “Kaufmann Assessment Battery for Children” (K-ABC) Melchers and Preuß, 2009; α = 0.74). For the central executive, we decided to use indirect measurements due to the insufficient reliability (α > 0.50) of the backward span task from the “Hamburg-Wechsler-Intelligence-Test for Children III” (HAWK-III) (Tewes et al., 1999). Indirect measurements of the central executive (e.g., Engel de Abreu et al., 2010) were derived from the framework of basic cognitive skills using the picture symbol test (NEPS-BZT; based on DST; Lang et al., 2007; α = 0.82) and the NEPS reasoning matrices test (NEPS-MAT; based on “raven-matrices”; Raven, 2009; α = 0.73). With the advantage of being non-verbal, these tests also demonstrated visual-storage capacities. We further used information on the socioeconomic status (SES) (based on ISEI-08; Ganzeboom, 2010), German as main domestic language (GERM) (0 = no/1 = yes), and sex (1 = male/2 = female) from parents’ and kindergarten/schoolteachers’ questionnaires.

### Data Analyses

Descriptive analyses were used to identify first between-group differences across all utilized variables. A more specific response to both research questions could be given by repeated measure ANCOVAs for mathematical skills [Weighted-Likelihood-Estimates (WLEs)] with the four groups (LD, MD, MD/LD, and TA) as between-subject factors. For linguistic skills (vocabulary, grammar), tests of mean differences were used due to the availability of separate sum scores. The separate sum scores of both linguistic tests were standardized in a previous step. Additionally, univariate ANCOVAs were computed for both domains mathematics and linguistics. Background variables (SES, GERM, and sex) as well as cognitive measurements (phonological loop, indirect measurements of the central executive) were included as covariates in repeated and univariate ANCOVAs. The direct measurements of the central executive (see above) acted as a control for the indirect measurement results (see Supplementary Materials 8, 9). In this paper direct findings will only be mentioned if they reveal any meaningful differences compared to the indirect measures used. To counteract an overestimation of group differences with respect to regression to the mean challenges (cf. Campbell and Kenny, 2003), all analyses were checked with slightly different cut-off points (see Supplementary Material 1). All described analyses were computed using SPSS 25.

## Results

### Descriptives

Initial information about group differences using descriptive statistics and univariate ANCOVAs or χ^2^-tests are summarized in Table 1. Significant differences between groups were found for all measurement time points for the linguistic and mathematical domains as well as the individual covariates, sex excluded. Notably, children with combined learning difficulties (MD/LD) received the lowest scores in all aforementioned measurements for both linguistic and mathematical skills. This group also scored lowest in all control variables (excluding sex).

**TABLE 1:** Descriptive statistics per group.

	MD (*n* = 26)	LD (*n* = 23)	MD/LD (*n* = 18)	TA (*n* = 234)	Total (*n* = 303)	
		
	*M (SD)*	*M (SD)*	*M (SD)*	*M (SD)*	*M (SD)*	*F*
Mathematics t1	−0.94 (0.08)	0.29 (0.12)	−1.24 (0.12)	0.77 (0.05)	0.47 (0.10)	151.75***
Mathematics t2	0.80 (0.16)	1.26 (0.19)	0.24 (0.24)	2.01 (0.07)	1.74 (1.12)	27.95***
Mathematics t3	1.49 (0.19)	2.10 (0.20)	1.19 (0.21)	2.70 (0.07)	2.46 (1.15)	20.50***
Mathematics t4	3.68 (0.22)	4.23 (0.18)	3.66 (0.22)	4.94 (0.07)	4.69 (1.12)	20.45***
Vocabulary t1	52.04 (1.54)	38.43 (2.32)	27.44 (2.85)	55.98 (0.40)	52.59 (10.68)	59.09***
Vocabulary t2	40.77 (1.41)	31.43 (1.86)	26.61 (1.28)	44.81 (0.44)	42.38 (8.69)	61.18***
Vocabulary t3	45.08 (1.60)	39.67 (1.61)	33.61 (1.79)	48.97 (0.50)	47.01 (8.59)	31.68***
Grammar t1	33.62 (0.61)	22.48 (1.13)	21.00 (1.38)	35.53 (0.28)	33.49 (6.43)	110.79***
Grammar t2	26.27 (1.08)	24.87 (1.05)	21.39 (1.18)	30.80 (0.30)	29.39 (5.46)	34.80***
Phonological loop	4.88 (0.39)	5.35 (0.39)	3.61 (0.39)	5.79 (0.12)	5.54 (1.95)	8.86***
Central executive	0.41 (0.02)	0.47 (0.02)	0.42 (0.02)	0.48 (0.01)	0.47 (0.13)	3.21*
SES	50.82 (3.87)	52.50 (4.43)	34.35 (4.13)	55.23 (1.21)	53.42 (18.68)	6.78***
	% (*n*)	% (*n*)	% (*n*)	% (*n*)	% (*n*)	χ^2^
GERM						52.58***
No	0.0 (0)	17.4 (4)	33.3 (6)	1.3 (3)	4.3 (13)	
Yes	100.0 (26)	82.6 (19)	66.7 (12)	98.7 (231)	95.7 (288)	
Sex						6.15
Male	53.8 (14)	56.5 (13)	22.2 (4)	48.3 (113)	47.8 (144)	
Female	46.2 (12)	43.5 (10)	77.8 (14)	51.3 (120)	51.8 (156)	

*Significant group differences are highlighted in bold.*

*SES = socioeconomic status; GERM = German as main domestic language.*

*p < 0.05, ***p < 0.001. MD/LD = children with combined learning difficulties in mathematics and linguistics; MD = children with mathematical learning difficulties; LD = children with linguistic learning difficulties; TA = typical achieving children.

Table 2 displays further detailed information about group differences in all competence measurements computed using Games-Howell *post-hoc* tests. Significant differences between all groups persisted only for time 1 of vocabulary, while the differences between children with mathematical learning difficulties (MD) and typically achieving peers (TA) only appeared when covariates were controlled for (without control of covariates: *p* = 0.085; under control of covariates without working memory: *p* = 0.022; under control of covariates incl. working memory: *p* = 0.087). For all other measurements of linguistic skills (vocabulary t2, t3; grammar t1, t2), *post-hoc* tests revealed no significant differences (*p* > 0.05) between the combined group (MD/LD) and the group with linguistic learning difficulties (LD). Additionally, at the last measurements of both linguistic skills (vocabulary t3, grammar t2), comparisons between both groups with domain-specific learning difficulties (LD and MD) bore no significant results (*p* > 0.05).

**TABLE 2:** Significant differences between groups w/o vs. under control of covariates computed by univariate ANCOVAs.

	MD/LD × MD	MD/LD × LD	MD/LD × TA	MD × LD	MD × TA	LD × TA
Mathematics t1		X	X	X	X	X
Mathematics t2		X^b^	X		X	X
Mathematics t3		X^ab^	X		X	X^ab^
Mathematics t4			X		X	X
Vocabulary t1	X	X^b^	X	X	X^c^	X
Vocabulary t2	X		X	X	X^ab^	X
Vocabulary t3	X^b^		X			X
Grammar t1	X		X	X	X^ab^	X
Grammar t2	X^b^		X		X	X
Phonological loop		X	X			
Central executive					X^a^	

*X = significant group differences computed by Games-Howell post-hoc tests.*

*a = non-significant under control of covariates w/o working memory.*

*b = non-significant under control of covariates w/ working memory.*

*c = only significant under control of covariates w/o working memory.*

*Significance level: p ≤ .05.*

*MD/LD = children with combined learning difficulties in mathematics and linguistics; MD = children with mathematical learning difficulties; LD = children with linguistic learning difficulties; TA = typical achieving children.*

Described for linguistic competencies, *post-hoc* tests revealed no significant differences (*p* > 0.05) for all measurements of mathematical skills between the group with combined difficulties (MD/LD) and the group with difficulties within the compared achievements (MD). Furthermore, no significant differences (*p* > 0.05) were found for mathematical skills between both groups with domain-specific learning difficulties (MD and LD), except for the measurement at pre-school age. For mathematical achievements in grade 4 (t2), we found no significant differences (*p* > 0.05) between the groups with combined learning difficulties (MD/LD) compared to linguistic learning difficulties (LD).

Working memory measurements at age 5/6, which were incorporated as additional covariates in the second part of the analyses of linguistic and mathematical competence development, showed only a few significant group deviations: For the phonological loop, *post-hoc* tests revealed differences (*p* ≤ 0.02) between the groups with combined difficulties (MD/LD) and linguistic difficulties (LD) as well as the combined group (MD/LD) and the typically achieving peers (TA) (*p* = 0.00). When compared for the central executive, scores of typical achievers (TA) and of children with mathematical learning difficulties (MD) differed only without control of the other covariates (without control of covariates: *p* < 0.05; under control of covariates: *p* = 0.15). Controlling for direct measurements of central executive by backward span tests showed additional significant group differences between the group with compared learning difficulties (MD/LD) and the typical achieving peers (TA) (*p* < 0.001) (see Supplementary Materials 8, 9).

### Mathematical and Linguistic Competence Development of Children With Different Learning Difficulties–Research Question 1

**FIGURE 1:**
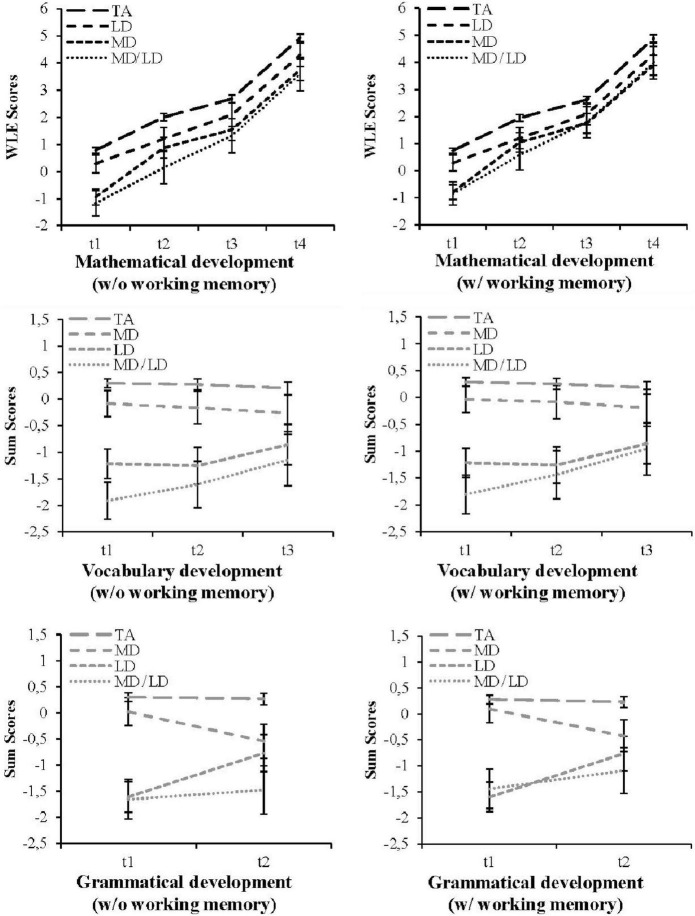
Development of mathematical (K-4), vocabulary (K-3), and grammar (K-1) skills in groups with different forms of pre-school measured learning difficulties (MD/LD: *n* = 18; MD: *n* = 26; LD: *n* = 23) vs. typically achieving children (TA: *n* = 234) under the control of covariates w/o vs. w/ working memory.

The left side of Figure 1 provides information to answer the first research question regarding *differences in mathematical and linguistic competence development between subgroups of children with domain-specific (MD, LD), combined (MD/LD), and without (TA) learning difficulties* under the control of individual characteristics used as covariates (GERM, SES, and sex).

#### Mathematical Development

Repeated measures ANCOVAs (see Figure 1; for a Supplementary Material 4) bared a significant interaction effect between mathematics and group (*F*(3, 804.69) = 2.46, *p* = 0.010, $\eta_{p}^{2}$ = 0.03; Greenhouse-Geisser corrected). The initial contribution of group (Table 1) for the individual measurement time points of mathematical competence development remains under control of covariates (GERM, SES, and sex). Children with combined learning difficulties showed the lowest values at each measurement time point, followed by children with mathematical learning difficulties (MD).

For covariates (GERM, SES, and sex), there was only a significant main (*F*(1, 257) = 18.26, *p* = 0.000, $\eta_{p}^{2}$ = 0.07) and interaction effect for the SES (mathematics × SES: *F*(3, 771) = 7.83, *p* = 0.000, $\eta_{p}^{2}$ = 0.03; Greenhouse-Geisser corrected), which also reflected univariate for the individual time points of mathematical skills in school age (Table 3).

**TABLE 3:** Univariates ANCOVAs of mathematical competences without vs. with working memory control.

	w/o working memory	w/ working memory	w/o working memory	w/ working memory	w/o working memory	w/ working memory	w/o working memory	w/ working memory
				
Mathematics	t1		t2		t3		t4	
	$\eta_{p}^{2}$ ***(F)***	$\eta_{p}^{2}$ ***(F)***	$\eta_{p}^{2}$ ***(F)***	$\eta_{p}^{2}$ ***(F)***	$\eta_{p}^{2}$ ***(F)***	$\eta_{p}^{2}$ ***(F)***	$\eta_{p}^{2}$ ***(F)***	$\eta_{p}^{2}$ ***(F)***

Model	0.43 (34.24***)	0.55 (42.03***)	0.29 (18.44***)	0.42 (23.96***)	0.21 (11.83***)	0.40 (22.12***)	0.25 (14.67***)	0.34 (17.27***)
Group	0.38 (56.27***)	0.35 (48.32***)	0.20 (23.08***)	0.15 (15.63***)	0.15 (15.67***)	0.10 (9.64***)	0.15 (15.95***)	0.11 (10.88***)
GERM	0.00 (0.32)	0.00 (1.19)	0.01 (3.46)	0.01 (2.54)	0.00 (0.83)	0.00 (0.25)	0.01 (2.15)	0.01 (1.47)
SES	0.00 (0.72)	0.01 (1.39)	0.06 (18.43***)	0.08 (21.96***)	0.03 (9.65**)	0.05 (13.51***)	0.09 (24.94***)	0.09 (25.76***)
Sex	0.01 (2.06)	0.04 (11.28***)	0.01 (2.71)	0.04 (11.47***)	0.01 (1.93)	0.04 (11.91***)	0.00 (0.03)	0.00 (0.97)
PL	–	0.03 (7.91**)	–	0.07 (18.69***)	–	0.05 (13.76***)	–	0.05 (14.73***)
CE	–	0.17 (55.56***)	–	0.09 (27.21***)	–	0.17 (54.43***)	-	0.06 (15.20***)

*Significant effects are highlighted in bold.*

*GERM = German as main-domestic language; SES = socioeconomic status; PL = phonological loop; CE = central executive.*

n = 303; **p ≤ 0.01 ***p ≤ 0.001.

Based on repeated measures, Figure 1 displays a nearly consistent mathematical development pattern of all groups until fourth grade. Besides, children with mathematical difficulties (MD) showed a short living improvement at school entry at measurement time 2. Further, the development of the group with combined difficulties (MD/LD) as well as merely mathematical difficulties (MD) achieved almost the same level between second and fourth grade (t2 until t4). A general lack of significant differences (*p* > 0.05) between these both groups in mathematics was already elaborated above in the reported Games-Howell *post-hoc* tests (Table 2).

#### Linguistic Development

As described in the method section, the analyses of the development of both linguistic competences (vocabulary, grammar) should be assessed by changes in group differences (see Supplementary Table 3) between each point in time due to the presence of non-longitudinally related linguistic data. The univariate ANCOVAs (see Table 4) already demonstrate strong main effects of groups (cf. Cohen, 1988: 0.01 = small effect, 0.06 = medium effect, 0.14 = large effect) for each model of the individual time points of both, vocabulary and grammar skills, under control of covariates. Like for mathematical development, the aforementioned hierarchical order between groups was also maintained in both linguistic skills (vocabulary, grammar): Children with combined learning difficulties (MD/LD) achieved always the lowest level, followed by children with linguistic learning difficulties (LD).

**TABLE 4:** Univariate ANCOVAs of linguistic competences without vs. with working memory control.

	w/o working memory	w/ working memory	w/o working memory	w/ working memory	w/o working memory	w/ working memory
			
Vocabulary	t1		t2		t3	
	$\eta_{p}^{2}$ ***(F)***	$\eta_{p}^{2}$ ***(F)***	$\eta_{p}^{2}$ ***(F)***	$\eta_{p}^{2}$ ***(F)***	$\eta_{p}^{2}$ ***(F)***	$\eta_{p}^{2}$ ***(F)***
Model	0.61 (72.12***)	0.62 (56.03***)	0.41 (31.65***)	0.43 (25.91***)	0.27 (16.83***)	0.30 (14.19***)
Group	0.46 (77.67***)	0.43 (68.88***)	0.31 (40.82***)	0.28 (35.98***)	0.16 (17.11***)	0.14 (13.82***)
GERM	0.09 (27.36***)	0.10 (29.27***)	0.00 (0.26)	0.00 (0.50)	0.02 (5.12*)	0.02 (6.18*)
SES	0.00 (0.53)	0.01 (1.60)	0.04 (11.55***)	0.04 (12.03***)	0.02 (4.18*)	0.02 (3.98*)
Sex	0.03 (8.16**)	0.04 (11.02***)	0.01 (1.39)	0.01 (3.47)	0.01 (1.37)	0.01 (3.11)
PL	-	0.01 (2.41)	-	0.01 (2.42)	-	0.02 (4.71*)
CE	-	0.01 (2.41)	-	0.02 (6.51*)	-	0.01 (2.73)

**Grammar**	**t1**		**t2**			
	**$\eta_{p}^{2}$** ***(F)***	**$\eta_{p}^{2}$ *(F)***	**$\eta_{p}^{2}$ *(F)***	**$\eta_{p}^{2}$ *(F)***		

Model	0.51 (48.37***)	0.54 (40.88***)	0.39 (23.55***)	0.43 (26.11***)		
Group	0.44 (72.53***)	0.42 (67.43***)	0.24 (28.80***)	0.20 (21.45***)		
GERM	0.00 (0.25)	0.00 (0.63)	0.00 (0.11)	0.00 (0.00)		
SES	0.02 (6.44*)	0.02 (6.14*)	0.05 (15.89***)	0.05 (15.22***)		
Sex	0.00 (0.05)	0.00 (1.14)	0.02 (6.05*)	0.01 (2.16)		
PL	-	0.04 (10.77***)	-	0.12 (36.73***)		
CE	-	0.02 (4.41*)	-	0.01 (2.50)		

*Significant effects are highlighted in bold.*

*GERM = German as main-domestic language; SES = socioeconomic status; PL = phonological loop; CE = central executive.*

n = 303; *p ≤ 0.05, **p ≤ 0.01, ***p ≤ 0.001.

The additional effects of the used covariates in univariate ANCOVAs (see Table 4), which already show an influence on the linguistic and mathematical competence level in pre-school age, indicated the need to control these: Analyses bared significant main effects of GERM at vocabulary skills in grade 3 (t3) as well as significant effects of SES at most points in time for both linguistic skills. As can be seen in Figure 1, the comparison of changes in mean differences between groups (see Supplementary Table 3) reveals a partial catch up of linguistic deficits for the group with pre-school measured linguistic difficulties (LD) in favor of grammar skills (vocabulary: LD × TA: t1: *diff* = 1.52, *SE*_*diff*_ = 0.11; t2: diff = 1.53, *SE*_*diff*_ = 0.12; t3: *diff* = 1.06; *SE*_*diff*_ = 0.13; grammar: LD × TA: t1: *diff* = 1.91, *SE*_*diff*_ = 0.11; t2: *diff* = 1.04, *SE*_*diff*_ = 0.13). Slightly lower and more pronounced in vocabulary skills, children with combined difficulties (MD/LD) showed also improvements in linguistic skills (vocabulary: MD/LD × TA: t1: *diff* = 2.21, *SE*_*diff*_ = 0.14; t2: *diff* = 1.88, *SE*_*diff*_ = 0.16; t3: *diff* = 1.35, *SE*_*diff*_ = 0.17; grammar: MD/LD × TA: t1: *diff* = 1.95, *SE*_*diff*_ = 0.16; t2: *diff* = 1.75, SEdiff = 0.16). In contrast, the linguistic development of children with mathematical difficulties (MD) draws a different picture: Mainly, grammatical skills experienced a significant slump in grade 1 (MD × TA: t1: *diff* = 0.28, *SE*_*diff*_ = 0.10; t2: *diff* = 0.81, *SE*_*diff*_ = 0.11), while vocabulary skills showed a slightly pronounced deterioration (MD × TA: t1: *diff* = 0.38, *SE*_*diff*_ = 0.10; t2: *diff* = 0.44, *SE*_*diff*_ = 0.11; t3: *diff* = 0.47, *SE*_*diff*_ = 0.12). The value of reported standard errors of mean differences can be attributed to the size of the compared subgroups (e.g., Morris, 2008).

### Mathematical and Linguistic Competence Development of Children With Different Learning Difficulties Under Control of Working Memory–Research Question 2

To answer the research question *to what extent the developmental differences between groups change when influences of working memory were controlled*, analyses were repeated with the addition of measurements of the phonological and central executive working memory. Considering Figure 1 as a whole, the mathematical and linguistic development processes under control of covariates w/o vs. w/ working memory could be compared. First of all, controlling phonological and central executive requirements minimized some group differences in favor of the group with combined difficulties: In mathematics, the initial gaps at the individual measurement time points decreased between the group with combined difficulties (MD/LD) and mathematical difficulties (MD). This pattern was identical in both linguistic skills between the groups with combined difficulties (MD/LD) and linguistic difficulties (LD). Additional decrease in group differences, which even changed the mean difference values from significant to non-significant level, emphasized the improvement of children with combined learning difficulties (MD/LD) at some more individual time points in the specific domains. These findings are reported in the following section on the development of the two specific domains.

#### Mathematical Development

In mathematics, the initial significant group differences between children with combined (MD/LD) and linguistic difficulties (LD) vanished in grades 1 and 2 (mathematics t2, t3) by controlling for both working memory components in addition to the described minimizations (MD/LD × MD) which hold the same level of significance (see also Table 2). However, besides a significant main effect of central executive (*F*(1, 254) = 54.64, *p* < 0.001, $\eta_{p}^{2}$ = 0.18) and phonological loop (*F*(1, 254) = 18.96, *p* < 0.001, $\eta_{p}^{2}$ = 0.07), repeated measurement ANCOVA revealed only a small significant mathematic × central executive interaction (*F*(3, 762) = 3.24, *p* = 0.023, $\eta_{p}^{2}$ = 0.01; Greenhouse-Geisser corrected). Further, univariate ANCOVAs (Table 3) showed a strongly increasing main effect of the models at each point in time under control of the phonological loop and the central executive in mathematics. For both working memory components, ANCOVAs also bared significant main effects for each time of mathematical measurements. In addition, there was a significant effect of sex for all measurements except in t4. Computing additional analyses (Supplementary Materials 5–7) rechecked the explanatory value of sex for the group differences of mathematics: Pearson’s bivariate correlations identified only significant relationships between sex and the central executive (sex × phonological loop: *r* = 0.09, *p* > 0.05; sex × central executive: *r* = 0.17, *p* ≤ 0.01). An univariate ANCOVA of the central executive with sex as group variable and phonological loop and SES as covariates (*F*(4, 285) = 6.94, *p* = 0.000, $\eta_{p}^{2}$ = 0.09) pointed to an explanatory value of group (*F*(4, 285) = 3.16, *p* = 0.044, $\eta_{p}^{2}$ = 0.02) in favor of female (male: *M* = 0.45; female: *M* = 0.48). Besides, descriptive analyses of the individual measurement time points per group (male/female) bared better achievements for male children at those measuring times of mathematical skills (uncorrected WLEs), which resulted in an explanatory value of sex under control of working memory (t1: *M* = 0.58, *SD* = 1.05/*M* = 0.36, *SD* = 0.95; t2: *M* = 1.85, *SD* = 1.15/*M* = 1.64, *SD* = 1.10; t3: *M* = 2.54, *SD* = 1.20/*M* = 2.39, *SD* = 1.11; t4: *M* = 4.67, *SD* = 1.13/*M* = 4.71, *SD* = 1.14).

#### Linguistic Development

Controlling for both working memory components also minimized some group differences in linguistic skills, in favor of children with combined learning difficulties (MD/LD). With the last measurements in vocabulary (t3) and grammar (t2) skills, the leveling between children with combined (MD/LD) and with mathematical difficulties (MD) even erased the level of significance (see Table 2). Vocabulary developments of the groups with combined (MD/LD) and linguistic (LD) difficulties are converging if working memory is controlled (c.f., Figure 1). Based on univariate ANCOVAs (Table 4), comparisons could be explained by a significant interaction between vocabulary and central executive in first grade (vocabulary t2: *F*(8, 275) = 6.51, *p* = 0.011, $\eta_{p}^{2}$ = 0.02) as well as phonological loop in third grade (vocabulary t3: *F*(8, 265) = 4.71, *p* = 0.031, $\eta_{p}^{2}$ = 0.02). For grammar skills, there were great differences of the influence of working memory control in pre-school and in primary-school age. In pre-school age (grammar t1), the control of working memory makes children with combined difficulties (MD/LD) perform even better than children with linguistic difficulties (LD). In this age, univariate ANCOVAs (Table 4) bared significant interaction effects between grammar skills and both, phonological loop (*F*(8, 275) = 10.77, *p* < 0.001, $\eta_{p}^{2}$ = 0.04) and central executive (*F*(8, 275) = 4.41, *p* = 0.037, $\eta_{p}^{2}$ = 0.02). In first grade (grammar t2), the effect of the working memory on group differences in grammar was solely caused by the phonological loop (grammar × phonological loop: *F*(8, 275) = 36.73, *p* < 0.001, $\eta_{p}^{2}$ = 0.12).

## Discussion

Using longitudinal data of the German National Educational Panel Study (NEPS), the present study sought to clarify the following interrelated issues under control of background variables: First, we wanted to investigate whether mathematical and linguistic development differs between subgroups of children with domain-specific, combined, and without any learning difficulties as identified in pre-school age. Furthermore, we were interested to which extent developmental differences between the groups changed when phonological and central executive working memory components were controlled.

Initially, children with combined difficulties had the worst performances for both mathematical and linguistic skills at any time, followed by children with difficulties in the examined competences. Comparable findings for the linguistic and mathematical development of children with specific, combined resp. without learning difficulties had already been published by Jordan and Hanich (2003), however, spanning the time period between second and third grade only. Controlling for covariates in our study, children with combined learning difficulties were also deprived in SES as well as the advantage of the main language of school instruction to be spoken at home. Research shows that both individual characteristics are strong predictors for lower performance in school in linguistics and mathematics (Demir et al., 2015; OECD, 2019). For the SES, our results confirmed these findings in the general model by explaining performance and development differences between groups for mathematical as well as linguistic skills, which is consistent with previous studies in children with learning difficulties (Stock et al., 2010; Dilnot et al., 2017). In contrast, the main language of school instruction being spoken at home only has an impact on the vocabulary skills of children, especially in pre-school age. This is consistent with results of large-scale development studies (e.g., TEDS, Plomin and Dale, 2001) which attribute the lesser input dependency for grammar vs. vocabulary skills on genetics.

### Mathematical Development

Considering mathematical development, a nearly consistent pattern was found between all groups from last year before school entry until the fourth grade of primary school with one small exception: Children with mere mathematical difficulties showed a short mathematical improvement at school entry respectively between age five to seven. This finding contrasts with the results of Jordan et al. (2015) who also examined the development of subgroups of children with domain-specific, combined and without learning difficulties of a comprehensive mathematical construct between 5 and 7 years, but with two significant differences: On the one hand, children within the study of Jordan et al. (2015) were already enrolled in school in the examined age span. On the other hand, in contrast to the unidimensional mathematical construct in NEPS, the study design allows a facet evaluation of mathematical skills. Nevertheless, Jordan et al. (2015) results did not show a different developmental thrust within this age range for any of the investigated mathematical task types. This indicates that our results of the leap in the development of children with low mathematical achievements could be explained by entering school. However, between first and second grade, children with low mathematical achievement fall repeatedly behind in their performances and show parallel progression to the other groups for the further development until the end of fourth grade. Apparently, it seems not possible to obtain a long-term support for the initially absorbed difficulties within the general framework of the school system. For the comparison of children with mathematical difficulties to their typical achieving peers, Geary et al. (2012) also have assessed a parallel development in similar school age, but especially for the skills of number sets. Further, results of the presented study showed no longer significant differences between the development of children with mathematical vs. combined difficulties in school, which was already proven for the arithmetic development in subtype comparison by Jordan and Hanich (2003).

### Linguistic Development

For linguistic development various patterns of changes in group differences between vocabulary and grammar skills were shown: Children with combined difficulties as well as merely linguistic difficulties caught up in both linguistic skills in comparison to their typical achieving peers, but in a different degree. In vocabulary skills, children with combined learning difficulties showed a clear catching-up between all points in time, while improvement of children with merely linguistic difficulties was weaker and only predominantly at school. In contrast, these children with the domain difficulties showed a strong jump at school entry in grammar skills, which was also shown in a much weaker form by children with combined difficulties. This finding may be related to the grammatical development, which, unlike the vocabulary, is completed at the age of 6–7 years (Markowitsch and Welzer, 2010). Comparing children with early language deficits and their control group, Rescorla (2002) also found these lower performance gaps for grammar than for vocabulary, strikingly for the age of six and seven.

Considering the linguistic development of children with low mathematical achievements, results revealed a contrasting picture: In both vocabulary and grammar skills, these children showed a decline compared to their typical achieving peers, in strong favor for grammar. Keeping in mind that both linguistic skills were measured as comprehension tests, comparisons can be drawn to Jordan and Hanich (2003) results: The authors found diverging developments in reading skills which were mainly assessed through comprehension tests (word identification, fluency, and passage comprehension). At least for grammar, our result could argue, with respect to Kleemans et al. (2012), to a joint principle of recursion for grammar and mathematics. Maybe we are talking here about cognitive abilities which are needed for a certain understanding of rules? In order to clarify such questions, the influence of cognitive abilities on the developmental processes and their different courses between the groups will be examined below.

### The Specific Role of Working Memory

Controlling for phonological and central executive requirements, the mathematical development between all groups showed smaller differences. Models of group-comparison for all mathematical competence measures bared a stronger explanatory value. Both, the phonological loop and the central executive can be used to explain group differences. These findings are consistent with the results of previous research which, for different components of working memory, show relationships with mathematical learning in general on the one hand and existing limitations in these components for children with mathematical learning difficulties on the other hand (for an overview: Peng and Fuchs, 2014). Moreover, the development of children with combined difficulties is improving in that, since second grade, it is almost identical to the development of children with only mathematical difficulties.

A marked improvement in children with combined difficulties under the control of working memory is also evident in linguistic development, especially in grammar. For vocabulary, the catching-up has a similar pattern as in mathematics. In grammatical development, pre-school performance under control of working memory is even better for children with combined than for children with only linguistic difficulties. Apparently, the capacity of working memory has a great explanatory value, notably for children with combined learning difficulties. This is in line with a current study of Brandenburg and Hasselhorn (2019), who were able to identify more comprehensive cognitive deficits, particularly in children with combined learning difficulties. Back to linguistic skills, we found a greater explanatory value of working memory for group-comparisons of grammar than for vocabulary which accords to our suggestion of underlying cognitive abilities for an understanding of rules. Further, in both linguistic skills, the central executive plays only a role for group differences in the early competence measurement, later replaced by the phonological loop. Maybe this is related to the results of Schuchardt et al. (2012). Studying the effects of both working memory components on the further development of children with linguistic difficulties in pre-school age, these authors found that persistent linguistic difficulties could only be explained by the continued existence of deficits in the phonological loop in school age.

### Limitations

Using a national panel dataset, some compromises had to be made regarding our own research interests: As not all data were evenly anchored to each other and competence scores had not been uniformly measured at all points in time, it was not possible to compute growth curve analyses which would have allowed to not only map the development of both skills–linguistic and math–simultaneously but also individually. Further, with growth curve modeling the study of interconnectedness in the development of linguistic and math skills would have been possible (e.g., Duncan and Duncan, 2009). Another problem of the NEPS data set refers to the lack of direct working memory measurements which forced us to use indirect data instead to operationalize the central executive. Although those indirect measures where evidenced in previous studies (e.g., Engel de Abreu et al., 2010), they need to be considered with care. It would be valuable for future research to more thoroughly address measuring working memory directly and in more detail. Finally, it was not possible to distinguish between the developmental patterns for specific mathematical skills as a unidimensional of the construct was applied by the NEPS consortium.

## Conclusion

The presented study helps to identify children with combined linguistic and mathematical learning difficulties in pre-school age as the most disadvantaged group for learning difficulties in further school development. The difficulties of these children go hand in hand with both, a reduced ability of the working memory as well as individual disadvantages due to their social background. With respect to research about home predictors on early literacy and numeracy (Kleemans et al., 2012) in addition to our findings we suggest addressing early inequalities as early as in pre-school age and continue intensively into primary-school age. Further, for a support as well as a prevention of limited working memory resources, approaches of working memory relieving teaching methods (e.g., Gathercole and Alloway, 2007) should be implemented extensively. Other existing approaches to a combined promotion of working memory and mathematical skills (Sanchez-Perrez et al., 2018) are in addition to a balance of memory-based weaknesses and the mathematical development, for which the lowest compensations could be reported for all forms of learning difficulties in our findings. For such promotion programs, even an additional effect on reading could be demonstrated. This linguistic ability also presupposes a certain understanding of rules such as grammar skills, which decreasing development in school is closely connected to mathematical and combined difficulties in the presented study. Further research is needed to detect the influences of such combination programs on grammatical understanding, especially in children with different learning difficulties. Finally, the earliest possible establishment of a combination of such described resource-oriented (e.g., Gathercole and Alloway, 2007) and resource-promoting (e.g., Sanchez-Perrez et al., 2018) approaches is considered to be promising in order to compensate for learning difficulties, both domain-specific and combined.

## Data Availability Statement

This study is based on data of the German National Educational Panel Study (NEPS). Data were prepared and disseminated to the scientific community by the Research Data Center at the Leibniz Institute for Educational Trajectories (RDC-LIfBi) (see https://www.neps-data.de/Data-Center/Data-Access).

## Ethics Statement

Written informed consent to participate in this study was provided by the participants’ legal guardian/next of kin. Available at: https://www.neps-data.de/Data-Center/Data-and-Documentation/Start-Cohort-Kindergarten/Documentation.

## Author Contributions

NV-N, UR, and WB: conception. NV-N and UR: formulation of the text parts. NV-N: literature research, data analysis, and discussion of results. All authors contributed to the article and approved the submitted version.

## Conflict of Interest

The authors declare that the research was conducted in the absence of any commercial or financial relationships that could be construed as a potential conflict of interest.

## Publisher’s Note

All claims expressed in this article are solely those of the authors and do not necessarily represent those of their affiliated organizations, or those of the publisher, the editors and the reviewers. Any product that may be evaluated in this article, or claim that may be made by its manufacturer, is not guaranteed or endorsed by the publisher.

## References

[B1] Aro T. Poikkeus A.-M. Eklund K. Tolvanen A. Laasko M.-L. Viholainen H. (2009). Effects of multidomain risk accumulation on cognitive, academic, and behavioural outcomes. *J. Clin. Child Adolesc. Psychol.* 38 883–898. 10.1080/15374410903258942 20183671

[B2] Baddeley A. (2012). Working memory: theories, models, and controversies. *Annu Rev. Psychol.* 63 1–29. 10.1146/annurev-psych-120710-100422 21961947

[B3] Baddeley A. D. (1986). *Working Memory.* Oxford: Clarendon Press.

[B4] Berendes K. Weinert S. Zimmermann S. Artelt C. (2013). Assessing language indicators across the lifespan within the German National Educational Panel Study (NEPS). *J. Educ. Res. Online* 5 15–49.

[B5] Bishop D. V. (1989). *TROG – Test for Reception of Grammar.* Abingdon: Thomas Leach Ltd.

[B6] Blossfeld H.-P. Roßbach H.-G. von Maurice J. (2011). Education as a lifelong process. [Special Issue]. *Zeitschrift Erziehungswissenschaft* 14 19–34. 10.1007/s11618-011-0179-2

[B7] Brandenburg J. Hasselhorn M. (2019). “A mixture modeling approach to profile cognitive functioning with learning disorders [Conference presentation],” in *Proceedings of the Joint conference of the Sections Educational Psychology and Developmental Psychology (paEpsy) of the German Psychological Society (DGPs)*, (Leipzig).

[B8] Campbell D. T. Kenny D. A. (2003). *A Primer on Regression Artifacts.* New York, NY: Guilford Press.

[B9] Cohen J. (1988). *Statistical Power Analysis for the Behavioral Sciences*, 2nd Edn. Hillsdale, NJ: Lawrence Erlbaum Associates, Publishers.

[B10] Demir ÖE. Prado J. Booth J. R. (2015). Parental socioeconomic status and the neural basis of arithmetic: differential relations to verbal and visuo-spatial representations. *Dev. Sci.* 18 1–16. 10.1111/desc.12268 25664675 PMC4522207

[B11] Dilnot J. Hamilton L. Maughan B. Snowling M. J. (2017). Child and environmental risk factors predicting readiness for learning in children at high risk of dyslexia. *Dev. Psychopathol.* 29 235–244. 10.1017/S0954579416000134 26900040 PMC5244446

[B12] Donker M. Kroesbergen E. Slot E. Van Viersen S. De Bree E. (2016). Alphanumeric and non-alphanumeric rapid automatized naming in children with reading and/or spelling difficulties and mathematical difficulties. *Learn Individ Diff.* 47 80–87. 10.1016/j.lindif.2015.12.011

[B13] Duncan T. E. Duncan S. C. (2009). The ABC’s of LGM: an introductory guide to latent variable growth curve modeling. *Soc. Pers. Psychol. Compass* 3 979–991. 10.1111/j.1751-9004.2009.00224.x 20577582 PMC2888524

[B14] Dunn L. M. Dunn D. M. (2007). *Peabody Picture Vocabulary Test (PPVT-4)*, 4th Edn. Upper Saddle River, NJ: Pearson.

[B15] Durkin K. Mok P. L. H. Conti-Ramsden G. (2013). Severity of specific language impairment predicts delayed development in number skills. *Front. Psychol.* 4:581. 10.3389/fpsyg.2013.00581 24027548 PMC3759789

[B16] Ebert S. Weinert S. (2013). “Predicting reading literacy in primary school: the contribution of various language indicators in preschool,” in *The Development of Reading Literacy From Early Childhood To Adolescence*, eds Pfost M. Artelt C. Weiner S. (Bamberg: University of Bamberg Press), 93–149.

[B17] Engel P. M. J. Santos F. H. Gathercole S. E. (2008). Are working memory measures free of socioeconomic influence? *J. Speech Lang. Hear. Res.* 51 1580–1587. 10.1044/1092-4388(2008/07-0210) 18695012

[B18] Engel de Abreu P. M. J. Conway A. R. A. Gathercole S. E. (2010). Working memory and fluid intelligence in young children. *Intelligence* 38 552–561. 10.1016/j.intell.2010.07.003

[B19] Fischbach A. Schuchardt K. Brandenburg J. Klesczewski J. Balke-Melcher C. Schmidt C. (2013). Prävalenz von lernschwächen und lernstörungen. Zur bedeutung der diagnosekriterien [Prevalence of learning weaknesses and learning disabilities. The significance of the diagnostic criteria]. *Lernen Lernstörungen* 2 65–76. 10.1024/2235-0977/a000035

[B20] Fletcher J. M. Lyonm G. R. Barnes M. Stuebing K. K. Francis D. J. Olson R. K. (2001). “Classification of learning disabilities: an evidence-based evaluation,” in *Identification of Learning Disabilities: Research to Practice*, eds Bradley R. Danielson L. Hallahan D. P. (Mahwah, NJ: Lawrence Erlbaum Associates Publishers), 185–250.

[B21] Fox A. V. (2006). *TROG-D Test zur Überprüfung des Grammatikverständnisses [TROG-D Test to Examine Grammar Comprehension].* Idstein: Schulz-Kirchner.

[B22] Ganzeboom H. B. G. (2010). “A new international socio-economic index [ISEI] of occupational status for the international standard classification of occupation 2008 [ISCO-08] constructed with data from the ISSP 2002-2007 [Conference presentation],” in *Proceedings of the Annual Conference of International Social Survey Programme*, (Lisbonl).

[B23] Garson G. D. (2015). *Missing Values Analysis and Data Imputation.* Charlotte, NC: Statistical Associates Publishing.

[B24] Gathercole S. E. Alloway T. P. (2007). *Understanding Working Memory. A Classroom Guide. London.* Available online at: https://www.mrc-cbu.cam.ac.uk/wp-content/uploads/2013/01/WM-classroom-guide.pdf (accessed December 14, 2021).

[B25] Geary D. C. Hoard M. K. Nugent L. Bailey D. H. (2012). Mathematical cognition in children with learning disabilities and persistent low achievement: a five-year prospective study. *J. Educ. Psychol.* 104 206–223. 10.1037/a0025398 27158154 PMC4855881

[B26] Hackman D. A. Betancourt L. M. Gallop R. Romer D. Brodsky N. L. Hurt H. (2014). Mapping the trajectory of socioeconomic disparity in working memory: parental and neighborhood factors. *Child Dev.* 85 1433–1445. 10.1111/cdev.12242 24779417 PMC4107185

[B27] Jordan J. A. Wylie J. Mulhern G. (2015). Mathematics and reading difficulty subtypes: minor phonological influences on mathematics for 5-7-years-old. *Front. Psychol.* 6:221. 10.3389/fpsyg.2015.00221 25798118 PMC4350393

[B28] Jordan N. C. Hanich L. B. (2003). Characteristics of children with moderate mathematics deficiencies: a longitudinal perspective. *Learn. Disabil. Res. Pract.* 18 213–221. 10.1111/1540-5826.00076

[B29] Kleemans J. Peeters M. Segers E. Verhoevan L. (2012). Child and home predictors of early numeracy skills in kindergarten. *Early Childhood Res. Q.* 27 471–477. 10.1016/j.ecresq.2011.12.004

[B30] Kucian K. von Aster M. (2015). Developmental dyscalculia. *Eur. J. Pediatr.* 174 1–13. 10.1007/s00431-014-2455-7 25529864

[B31] Kühn P. (2010). *Wie Entwickeln sich Late Talkers? Eine Längsschnittstudie zur Prognose der Sprachlichen, Kognitiven und Emotionalen Entwicklung von Late Talkers bis zum Einschulungsalter [How do Late Talkers develop? A Longitudinal Study to Predict the Linguistic, Cognitive and Emotional Development of Late Talkers Until School Age].* München: Hut.

[B32] Kwok F. Y. Ansari D. (2019). The promises of educational neuroscience: examples from literacy and numeracy. *Learn. Res. Pract.* 5 189–200. 10.1080/23735082.2019.1677405

[B33] Lang F. R. Weiss D. Stocker A. von Rosenbladt B. (2007). Assessing cognitive capacities in computer-assisted survey research: two ultra-short tests of intellectual ability in the German Socio-Economic Panel (SOEP). *J. Appl. Soc. Sci. Stud.* 127 183–192.

[B34] Lewis A. (1980). The early identification of children with learning difficulties. *J. Learn. Disabil.* 13 102–108. 10.1177/002221948001300210 7391673

[B35] Markowitsch H. J. Welzer H. (2010). *The Developmental of Autobiographical Memory.* Hove: Psychology Press.

[B36] Melchers P. Preuß U. (2009). *Kaufmann Assessment Battery for Children (K-ABC)*, 8th Edn. Frankfurt: Pearson.

[B37] Moll K. Göbel S. M. Gooch D. Landerl K. Snowling M. J. (2014). Cognitive risk factors for specific learning disorder: processing speed, temporal processing, and working memory. *J. Learn. Disabil.* 49 272–281. 10.1177/0022219414547221 25124507

[B38] Morris S. B. (2008). Estimating effect sizes from pretest-posttest-control group designs. *Organ. Res. Methods* 11 364–386. 10.1177/1094428106291059

[B39] Neumann I. Duchhardt C. Grüßing M. Heinze A. Knopp E. Ehmke T. (2013). Modeling and assessing mathematical competence over the lifespan. *J. Educ. Res. Online* 5 80–109.

[B40] OECD (2013). *PISA 2012 Assessment and Analytical Framework: Mathematics, Reading, Science, Problem Solving and Financial Literacy.* Paris: OECD Publishing, 10.1787/9789264190511-en

[B41] OECD (2019). *PISA 2018 results (Volume II). Where all the Students can Succeed.* Paris: OECD Publishing, 10.1787/b5fd1b8f-en

[B42] Peng P. Fuchs D. (2014). A Meta-Analysis of working memory deficits in children with learning difficulties: is there a difference between verbal domain and numerical domain? *J. Learn. Disabil.* 49 3–20. 10.1177/0022219414521667 24548914

[B43] Peng P. Wang C. Namkung J. (2018). Understanding the cognition related to mathematics difficulties: a meta-analysis on the cognitive deficit profiles and the bottleneck theory. *Rev. Educ. Res.* 88 434–476. 10.3102/0034654317753350

[B44] Peters L. Bulthé J. Daniels N. Op de Beeck H. De Smedt B. (2018). Dyscalculia and dyslexia: different behavioral, yet similar brain activity profiles during arithmetic. *NeuroImage* 18 663–674. 10.1016/j.nicl.2018.03.003 29876258 PMC5987869

[B45] Plomin R. Dale P. S. (2001). “Genetics and early language development: a UK study of twins,” in *Speech and Language Impairments in Children: Causes, Characteristics, Intervention and Outcome*, eds Bishop D. V. M. Leonard L. B. (Hove: Psychology Press), 35–51.

[B46] Raven J. C. (2009). *Raven’s Progressive Matrices and Vocabulary Scales (SPM).* Göttingen: Hogrefe.

[B47] Rescorla L. (2002). Language and reading outcomes to age 9 in late-talking toddlers. *J. Speech Lang. Hear. Res.* 45 360–371. 10.1044/1092-4388(2002/028) 12003517

[B48] Sanchez-Perrez N. Castillo A. Lopez-Lopez J. A. Pina V. Puga J. L. Campoy G. (2018). Computer-based training in math and working memory improves cognitive skills and academic achievement in primary school children: behavioral results. *Front. Psychol.* 8:2327. 10.3389/fpsyg.2017.02327 29375442 PMC5767320

[B49] Sarsour K. Sheridan M. Douglas J. Nuru-Jeter A. Hinshaw S. (2010). Family socioeconomic status and child executive functions: the roles of language, home environment, and single parenthood. *J. Int. Neuropsychol. Soc.* 17 120–132. 10.1017/S1355617710001335 21073770

[B50] Schuchardt K. Worgt M. Hasselhorn M. (2012). “Besonderheiten im arbeitsgedächtnis bei kindern mit sprachauffälligkeiten [Special features of working memory in children with speech disorders],” in *Funktionsdiagnostik des Arbeitsgedächtnisses [Functional Diagnostic of the Working Memory]*, eds Hasselhorn M. Zoelch C. (Göttingen: Hogrefe), 77–93. 10.1007/978-3-642-73937-8_5

[B51] Snowling M. J. Adams J. W. Bishop D. V. M. Stothard S. E. (2001). Educational attainments of school leavers with a preschool history of speech-language impairments. *Int. J. Lang. Commun. Dis.* 36 173–183. 10.1080/13682820120976 11344593

[B52] Snowling M. J. Duff F. J. Nash H. M. Hulme C. (2016). Language profiles and literacy outome of children with resolving, emerging, or persisting language impairments. *J. Child Psychol. Psychiatry* 57 1360–1369. 10.1111/jcpp.12497 26681150 PMC5132029

[B53] Stock P. Desoete A. Roeyers H. (2010). Detecting children with arithmetic disabilities from kindergarten: evidence from a 3-year longitudinal study on the role of preparatory arithmetic abilities. *J. Learn. Disabil.* 43 250–268. 10.1177/0022219409345011 19903867

[B54] Stothard S. E. Hulme C. (1992). Reading comprehension difficulties in children. The role of language comprehension and working memory skills. *Read. Writ.* 4 245–256. 10.1007/bf01027150

[B55] Tewes U. Rossmann P. Schallberger U. (1999). *Hamburg-Wechsler-Intelligenztest für Kinder III (HAWIK-III).* Bern: Huber Verlag.

[B56] Viesel-Nordmeyer N. Reuber J. Kuhn T. Moll K. Holling H. Dobel C. (Submitted). *The Relation Between Cognitive Profiles of Children With Isolated and Comorbid Learning Difficulties in Reading and Math: a Meta-Analysis.*

