# Supplementary material for: Acquisition of Mathematical and Linguistic Skills in Children With Learning Difficulties
Source: Front Psychol. 2021 Dec 28;12:793796. doi: 10.3389/fpsyg.2021.793796 (PMC8751487; doi:10.3389/fpsyg.2021.793796)
Supplement: Supplementary file 1 [file Data_Sheet_1.PDF]

Supplementary Material

This online supplemental material contains further details on:

- **Section 1.** Checkup of all analyses with groups based on divergent cut-off points
- **Section 2.** Mean differences between the group values of individual competence measurements
- **Section 3.** Repeated measures ANCOVA of mathematical development with gradual addition of the covariates
- **Section 4.** Check of the explanatory value of sex for group differences in mathematics
- **Section 5.** Comparison of analyses with the initial central executive measurement vs. the utilized central executive measurement

Section 1. Checkup of all analyses with groups based on divergent cut-off points

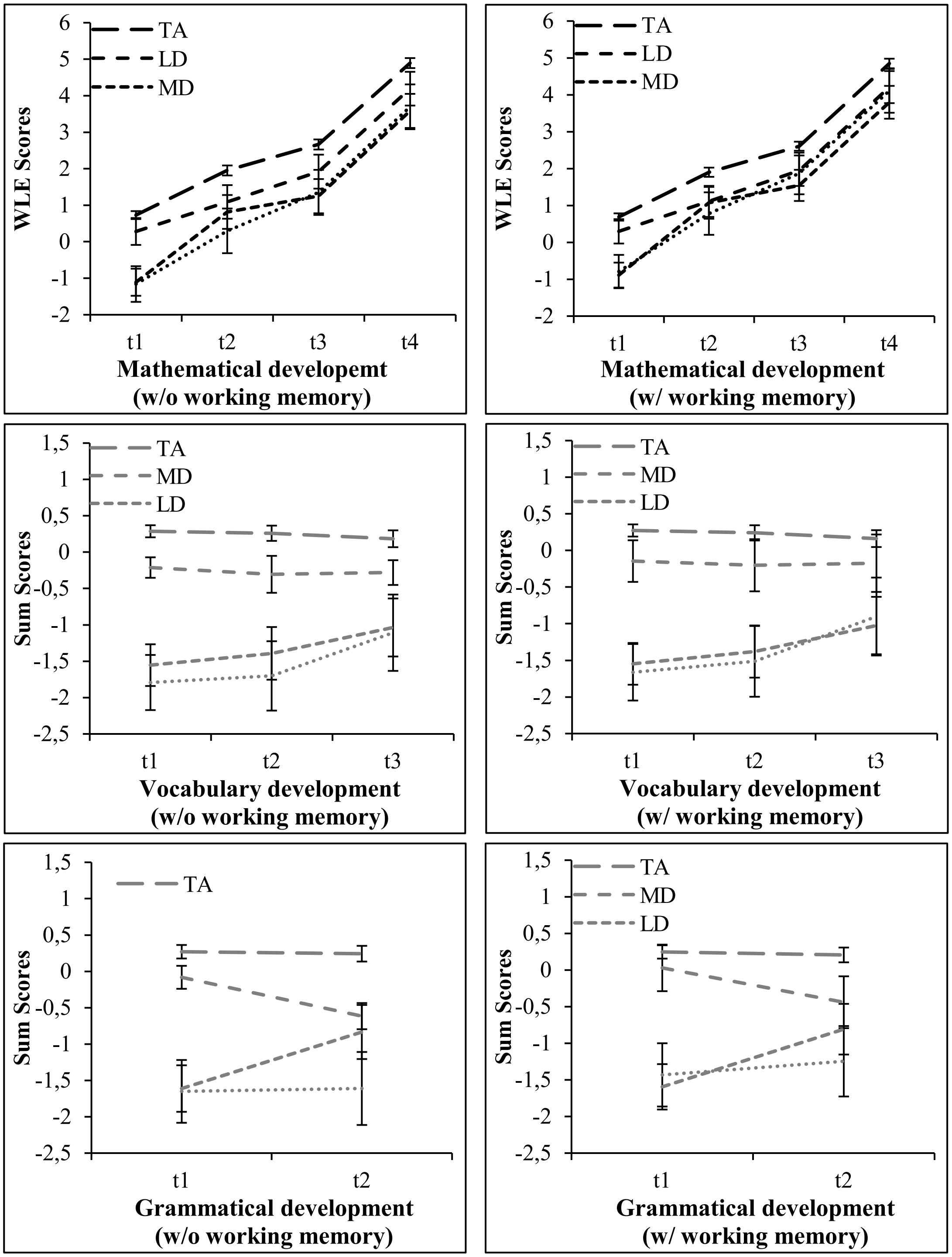

Supplementary Figure 1. Development of mathematical (K-4), vocabulary (K-3), and grammar (K-1) skills in groups (cut-off point -1.1 *SD*) with different forms of pre-school measured learning difficulties (MD/LD: n = 15; MD: n = 20; LD: n = 22) vs. typically achieving children (TA: n = 244) under the control of covariates w/o vs. w/ working memory

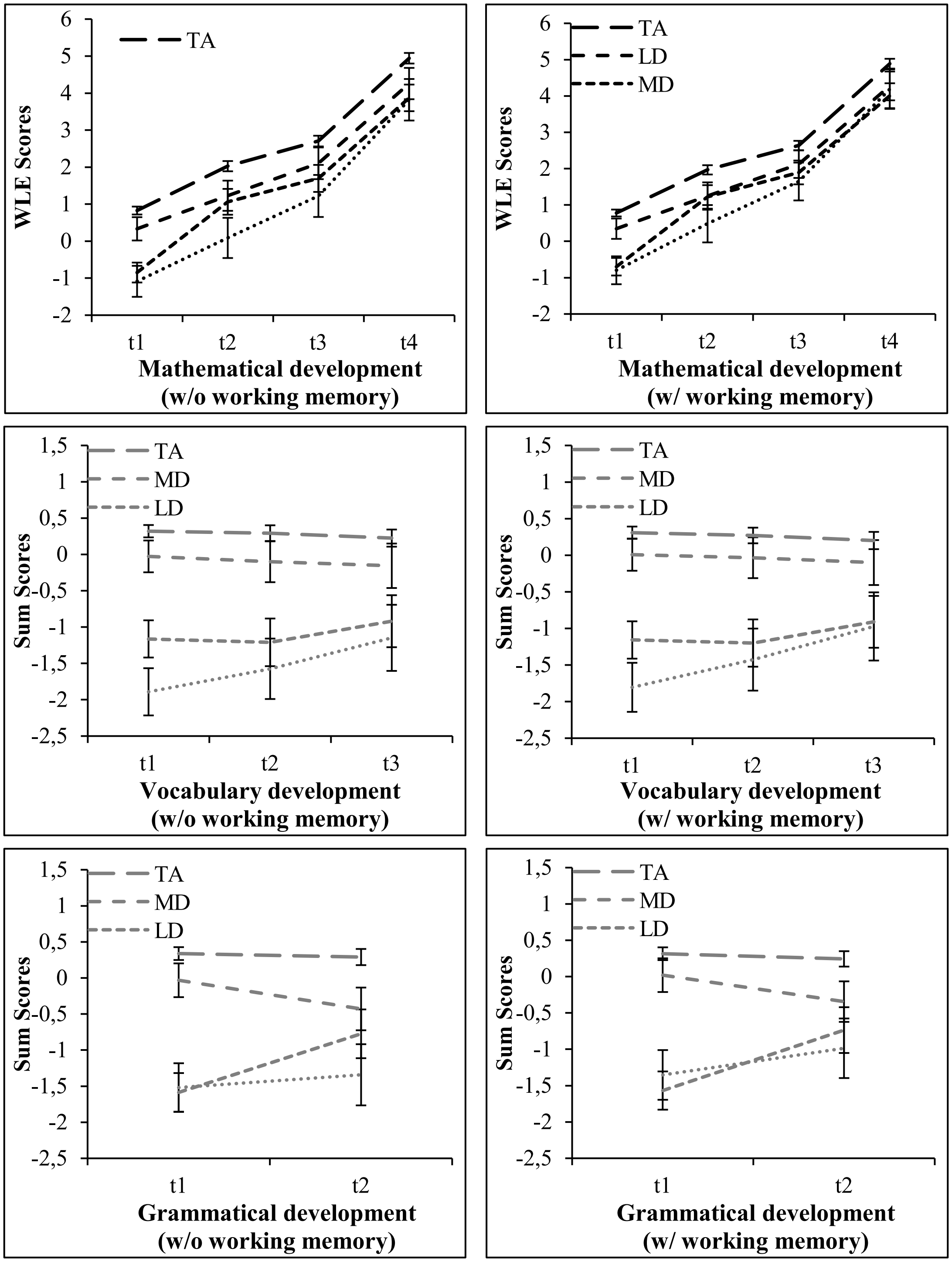

Supplementary Figure 2. Development of mathematical (K-4), vocabulary (K-3), and grammar (K-1) skills in groups (cut-off point -0.9 *SD*) with different forms of pre-school measured learning difficulties (MD/LD: n = 21; MD: n = 32; LD: n = 24) vs. typically achieving children (TA: n = 224) under the control of covariates w/o vs. w/ working memory.

Supplementary Table 1. *Differences between the group values (cut-off point -1.1 SD) of individual competence measurements under the control of covariates with vs. without working memory control*

	MD/LD*MD		MD/LD*LD		MD/LD*TA		MD*LD		MD*TA		LD*TA	
	diff (SE_{diff})		*diff (SE_{diff})*		*diff (SE_{diff})*		*diff (SE_{diff})*		*diff (SE_{diff})*		*diff (SE_{diff})*	
	working memory		working memory		working memory		working memory		working memory		working memory	
	w/o	w/	w/o	w/	w/o	w/	w/o	w/	w/o	w/	w/o	w/
Mathematics												
t1	0.05<br>(0.22)	0.10<br>(0.21)	**1.44<br>(0.23)**	**1.09<br>(0.22)**	**1.89<br>(0.18)**	**1.48<br>(0.17)**	**1.39<br>(0.19)**	**1.19<br>(0.18)**	**1.84<br>(0.13)**	**1.58<br>(0.13)**	0.45<br>(0.14)	0.39<br>(0.13)
t2	0.52<br>(0.25)	0.29<br>(0.24)	0.79<br>(0.25)	0.33<br>(0.24)	**1.66<br>(0.20)**	**1.12<br>(0.20)**	0.28<br>(0.21)	0.04<br>(0.20)	**1.14<br>(0.15)**	**0.83<br>(0.14)**	**0.86<br>(0.15)**	**0.79<br>(0.15)**
t3	0.10<br>(0.25)	0.32<br>(0.24)	0.57<br>(0.25)	0.08<br>(0.24)	**1.31<br>(0.20)**	**0.74<br>(0.20)**	0.67<br>(0.21)	0.40<br>(0.20)	**1.42<br>(0.15)**	**1.06<br>(0.14)**	**0.74<br>(0.15)**	**0.66<br>(0.15)**
t4	0.11<br>(0.25)	0.31<br>(0.24)	0.50<br>(0.25)	0.10<br>(0.25)	**1.20<br>(0.20)**	**0.74<br>(0.20)**	0.61<br>(0.21)	0.41<br>(0.20)	**1.31<br>(0.15)**	**1.05<br>(0.14)**	**0.70<br>(0.16)**	0.64<br>(0.15)
Vocabulary												
*t*1	**1.58<br>(0.18)**	**1.52<br>(0.20)**	0.24<br>(0.20)	0.12<br>(0.20)	**2.08<br>(0.16)**	**1.94<br>(0.16)**	**1.34<br>(0.14)**	**1.40<br>(0.16)**	**0.50<br>(0.08)**	0.42<br>(0.12)	**1.84<br>(0.12)**	**1.82<br>(0.12)**
t2	**1.40<br>(0.21)**	**1.31<br>(0.22)**	0.31<br>(0.22)	0.13<br>(0.22)	**1.96<br>(0.18)**	**1.75<br>(0.18)**	**1.09<br>(0.17)**	**1.18<br>(0.18)**	**0.57<br>(0.11)**	0.44<br>(0.13)	**1.65<br>(0.14)**	**1.62<br>(0.14)**

t3	**0.83 (0.21)**	**0.73 (0.23)**	0.07 (0.23)	0.12 (0.23)	**1.29 (0.19)**	**1.06 (0.19)**	**0.76 (0.17)**	**0.85 (0.19)**	0.47 (0.09)	0.34 (0.14)	**1.22 (0.14)**	**1.19 (0.14)**
Grammar												
t1	1.57 (0.19)	1.46 (0.21)	**0.04 (0.21)**	**0.16 (0.21)**	**1.92 (0.17)**	**1.68 (0.17)**	**1.53 (0.15)**	**1.62 (0.17)**	0.35 (0.09)	0.22 (0.12)	**1.88 (0.13)**	**1.84 (0.13)**
t2	**1.00 (0.20)**	0.81 (0.22)	0.78 (0.23)	0.44 (0.22)	**1.85 (0.18)**	**1.45 (0.18)**	0.22 (0.16)	0.37 (0.18)	**0.86 (0.09)**	**0.65 (0.13)**	**1.08 (0.14)**	**1.01 (0.13)**

Notes. Significant differences ($p < .05$) between group values of the individual competence measurements are in bold. MD/LD = children with combined learning difficulties in mathematics and linguistics (n = 15); MD = children with mathematical learning difficulties (n = 20); LD = children with linguistic learning difficulties (n = 22); TA = typical achieving children (n = 244).

Supplementary Table 2. *Differences between the group values (cut-off point -.9 SD) of individual competence measurements under the control of covariates with vs. without working memory control*

	MD/LD*MD		MD/LD*LD		MD/LD*TA		MD*LD		MD*TA		LD*TA	
	diff (SE_{diff})		*diff* (SE_{diff})		*diff* (SE_{diff})		*diff* (SE_{diff})		*diff* (SE_{diff})		*diff* (SE_{diff})	
	working memory		working memory		working memory		working memory		working memory		working memory	
	w/o	w/	w/o	w/	w/o	w/	w/o	w/	w/o	w/	w/o	w/
Mathematics												
t1	0.24	0.10	**1.45**	**1.14**	**1.91**	**1.58**	**1.18**	**1.05**	**1.68**	**1.48**	**0.49**	0.43
	(0.18)	(0.17)	**(0.17)**	**(0.16)**	**(0.14)**	**(0.14)**	**(0.15)**	**(0.14)**	**(0.11)**	**(0.10)**	**(0.10)**	(0.10)
t2	**0.98**	0.74	**0.80**	0.76	**1.94**	**1.48**	0.16	0.02	**0.96**	**0.75**	**0.80**	**0.72**
	**(0.20)**	(0.19)	**(0.20)**	(0.19)	**(0.16)**	**(0.16)**	(0.17)	(0.16)	**(0.13)**	**(0.12)**	**(0.12)**	**(0.11)**
t3	0.47	0.48	**0.88**	0.25	**1.48**	**0.99**	0.41	0.23	**1.01**	**0.74**	**0.60**	0.51
	(0.21)	(0.20)	**(0.20)**	(0.19)	**(0.17)**	**(0.16)**	(0.17)	(0.16)	**(0.13)**	**(0.12)**	**(0.12)**	(0.11)
t4	0.05	0.18	0.44	0.09	**1.12**	**0.70**	0.39	0.27	**1.07**	**0.88**	**0.68**	**0.61**
	(0.20)	(0.20)	(0.20)	(0.20)	**(0.17)**	**(0.16)**	(0.17)	(0.16)	**(0.13)**	**(0.12)**	**(0.12)**	**(0.11)**
Vocabulary												
*t*1	**1.86**	**1.81**	**0.73**	**0.65**	**2.21**	**2.11**	**1.14**	**1.17**	0.35	**0.30**	**1.48**	**1.46**
	**(0.16)**	**(0.16)**	**(0.15)**	**(0.15)**	**(0.13)**	**(0.13)**	**(0.13)**	**(0.13)**	(0.10)	**(0.10)**	**(0.09)**	**(0.09)**
t2	**1.47**	**1.46**	0.36	0.23	**1.87**	**1.70**	**1.11**	**1.17**	**0.39**	0.30	**1.50**	**1.47**
	**(0.18)**	**(0.18)**	(0.17)	(0.17)	**(0.14)**	**(0.14)**	**(0.15)**	**(0.15)**	**(0.11)**	(0.11)	**(0.10)**	**(0.10)**

t3	**0.99 (0.19)**	**0.96 (0.19)**	0.23 (0.18)	0.06 (0.18)	**1.37 (0.15)**	**1.17 (0.15)**	**0.76 (0.15)**	**0.81 (0.15)**	0.38 (0.12)	0.30 (0.12)	**1.14 (0.11)**	**1.11 (0.11)**
Grammar												
t1	**1.49 (0.16)**	**1.37 (0.16)**	0.07 (0.16)	0.22 (0.16)	**1.86 (0.13)**	**1.67 (0.13)**	**1.55 (0.13)**	**1.59 (0.13)**	**0.37 (0.10)**	0.29 (0.10)	**1.92 (0.09)**	**1.88 (0.09)**
t2	**0.91 (0.18)**	0.64 (0.18)	**0.57 (0.18)**	0.25 (0.17)	**1.63 (0.14)**	**1.23 (0.14)**	0.35 (0.15)	0.39 (0.15)	**0.72 (0.11)**	**0.59 (0.11)**	**1.06 (0.11)**	**0.98 (0.10)**

Notes. Significant differences ($p < .05$) between group values of the individual competence measurements are in bold. MD/LD = children with combined learning difficulties in mathematics and linguistics (n = 21); MD = children with mathematical learning difficulties (n = 24); LD = children with linguistic learning difficulties (n = 32); TA = typical achieving children (n = 224).

2 Mean-differences between the group values of individual competence measurements

Supplementary Table 3. *Differences between the group values of individual competence measurements under the control of covariates w/o vs. with working memory control*

	MD/LD*MD		MD/LD*LD		MD/LD*TA		MD*LD		MD*TA		LD*TA	
	diff (SE_{diff})		*diff (SE_{diff})*		*diff (SE_{diff})*		*diff (SE_{diff})*		*diff (SE_{diff})*		*diff (SE_{diff})*	
	working memory		working memory		working memory		working memory		working memory		working memory	
	w/o	w/	w/o	w/	w/o	w/	w/o	w/	w/o	w/	w/o	w/
Mathematics												
t1	0.23	0.06	**1.46**	**1.13**	**1.95**	**1.59**	**1.23**	**1.07**	**1.72**	**1.53**	**0.49**	0.46
	(0.20)	(0.19)	**(0.20)**	**(0.19)**	**(0.16)**	**(0.16)**	**(0.16)**	**(0.15)**	**(0.11)**	**(0.10)**	**(0.12)**	(0.12)
t2	0.73	0.47	**1.06**	0.62	**1.86**	**1.37**	0.33	0.15	**1.13**	**0.90**	**0.80**	**0.75**
	(0.22)	(0.21)	**(0.23)**	(0.22)	**(0.18)**	**(0.18)**	(0.18)	(0.17)	**(0.12)**	**(0.12)**	**(0.14)**	**(0.13)**
t3	0.23	0.04	0.77	0.29	**1.36**	**0.82**	0.55	0.34	**1.13**	**0.87**	**0.58**	0.53
	(0.22)	(0.21)	(0.23)	(0.22)	**(0.19)**	**(0.18)**	(0.19)	(0.18)	**(0.13)**	**(0.12)**	**(0.14)**	(0.13)
t4	0.15	0.09	0.70	0.31	**1.34**	**0.90**	0.55	0.40	**1.18**	**0.99**	**0.64**	**0.40**
	(0.22)	(0.22)	(0.23)	(0.23)	**(0.19)**	**(0.18)**	(0.18)	(0.18)	**(0.13)**	**(0.12)**	**(0.14)**	**(0.13)**
Vocabulary												
*t*1	**1.83**	**1.77**	**0.70**	**0.59**	**2.21**	**2.09**	**1.13**	**1.18**	**0.38**	**0.33**	**1.52**	**1.50**
	**(0.17)**	**(0.17)**	**(0.18)**	**(0.18)**	**(0.14)**	**(0.14)**	**(0.15)**	**(0.15)**	**(0.10)**	**(0.10)**	**(0.11)**	**(0.11)**
t2	**1.45**	**1.35**	0.36	0.18	**1.88**	**1.69**	**1.09**	**1.17**	**0.44**	0.34	**1.53**	**1.51**
	**(0.19)**	**(0.19)**	(0.20)	(0.20)	**(0.16)**	**(0.16)**	**(0.16)**	**(0.16)**	**(0.11)**	(0.11)	**(0.12)**	**(0.12)**

t3	**0.88 (0.20)**	**0.76 (0.20)**	0.29 (0.21)	0.10 (0.21)	**1.35 (0.17)**	**1.13 (0.17)**	0.59 (0.17)	0.67 (0.17)	0.47 (0.12)	0.37 (0.12)	**1.06 (0.13)**	**1.04 (0.13)**
Grammar												
t1	**1.86 (0.18)**	**1.54 (0.18)**	0.05 (0.18)	0.16 (0.18)	**1.95 (0.16)**	**1.72 (0.15)**	**1.63 (0.15)**	**1.70 (0.15)**	0.28 (0.10)	0.18 (0.10)	**1.91 (0.11)**	**1.88 (0.11)**
t2	**0.93 (0.20)**	0.67 (0.19)	0.70 (0.20)	**0.33 (0.20)**	**1.75 (0.16)**	**1.32 (0.16)**	0.23 (0.17)	0.34 (0.16)	**0.81 (0.11)**	**0.65 (0.11)**	**1.04 (0.13)**	**0.99 (0.12)**

Notes. Significant differences ($p < .05$) between group values of the individual competence measurements are in bold. MD/LD = children with combined learning difficulties in mathematics and linguistics (*n* = 18); MD = children with mathematical learning difficulties (*n* = 26); LD = children with linguistic learning difficulties (*n* = 23); TA = typical achieving children (*n* = 234).

3 Repeated measures ANCOVA of mathematical development with gradual addition of the covariates

Supplementary Table 4. *Repeated measures ANCOVA of mathematical development with gradual addition of the covariates*

	η_p^2 *(F)*	η_p^2 *(F)*	η_p^2 *(F)*	η_p^2 *(F)*	η_p^2 *(F)*	η_p^2 *(F)*	η_p^2 *(F)*
Mathematic	**.88 (2111.41***)**	**.76 (871.53***)**	**40. (169.58***)**	**.23 (77.06***)**	**.13 (37.74***)**	**.12 (34.86***)**	**.11 (31.00***)**
Mathematic*Group	–	**.01 (2.46**)**	**.04 (3.22***)**	**.04 (3.12***)**	**.03 (2.63**)**	**.03 (2.66**)**	**.03 (2.91**)**
Mathematic*SES	–	–	**.03 (6.96***)**	**.03 (6.61***)**	**.03 (7.47***)**	**.03 (7.40***)**	**.03 (6.71***)**
Mathematic*Sex	–	–	–	.01 (1.85)	.01 (1.83)	.01 (1.67)	.01 (1.58)
Mathematic*GMDL	–	–	–	–	.01 (1.78)	.01 (2.03)	.01 (2.87)
Mathematic*CE						**.01 (3.11*)**	**.01(3.24*)**
Mathematic*PL							.01 (2.13)

Notes. Repeated measures ANCOVA. Significant effects are highlighted in bold. SES = socioeconomic status; GMDL = German as main domestic language; CE = central executive; PL = phonological loop. n = 303; *= $p \leq .05$, ** = $p \leq .01$, *** = $p \leq .001$; n = 303.

4 Check of the explanatory value of sex for group differences in mathematics

Supplementary Table 5. *Pearson correlations between working memory components and sex*

	1	2
1 Phonological loop		
2 Central executive	**.27****	
3 Sex	.09	.17**

Notes. Values printed in bold represent significant relationships, *= $p \leq .05$, ** = $p \leq .01$; $n = 303$.

Supplementary Table 6. *Univariate ANCOVA of central executive with sex as group variable*

Central executive	**(F(4, 285) = 6.94, p < .001, η_n^2 = .09)**
Sex	**(F(4, 285) = 3.16, p = .044, η_n^2 = .02)**
Phonological loop	**(F(4, 285) = 18.75, p < .001, η_n^2 = .06)**
SES	(F(4, 285) = .89, p = .346, η_n^2 = .01)

Notes. n = 303; SES = socioeconomic status.

Supplementary Table 7. *Descriptive overview of mathematical competencies between male and female children*

	male (*n* = 156)		female (*n* = 146)	
	M	*SD*	*M*	*SD*
Mathematic t1	0.58	1.05	0.36	0.95
Mathematic t2	1.85	1.15	1.64	1.10
Mathematic t2	2.54	1.20	2.39	1.11
Mathematic t3	4.67	1.13	4.71	1.14

5 Comparison of analyses with the initial central executive measurement vs. the utilized central executive measurement

Supplementary Table 8. *Univariate ANCOVAs of mathematical competencies with direct vs. indirect measurement of central executive*

	central executive (direct)	central executive (indirect)	central executive (direct)	central executive (indirect)	central executive (direct)	central executive (indirect)	central executive (direct)	central executive (indirect)
Mathematics	t1		t2		t3		t4	
	η^2_p *(F)*	η^2_p *(F)*	η^2_p *(F)*	η^2_p *(F)*	η^2_p *(F)*	η^2_p *(F)*	η^2_p *(F)*	η^2_p *(F)*
Model	**.50 (33.87***)**	**.55 (42.03***)**	**.38 (20.52***)**	**.42 (23.96***)**	**.32 (15.83***)**	**.40 (22.12***)**	**.31 (14.59***)**	**.34 (17.27***)**
Group	**.30 (38.21***)**	**.35 (48.32***)**	**.12 (12.69***)**	**.15 (15.63***)**	**.07 (7.02***)**	**.10 (9.64***)**	**.10 (9.42***)**	**.11 (10.88***)**
GERM	.00 (.78)	.00 (1.19)	.01 (2.14)	.01 (2.54)	.00 (.08)	.00 (.25)	.01 (2.61)	.01 (1.47)
SES	.00 (.01)	.01 (1.39)	**.05 (15.26***)**	**.08 (21.96***)**	**.03 (6.83**)**	**.05 (13.51***)**	**.07 (20.94***)**	**.09 (25.76***)**
Sex	**.02 (5.14*)**	**.04 (11.28***)**	**.03 (6.76**)**	**.04 (11.47***)**	**.02 (5.33*)**	**.04 (11.91***)**	.00 (.41)	.00 (.97)
PL	**.03 (7.84**)**	**.03 (7.91**)**	**.07 (18.71***)**	**.07 (18.69***)**	**.05 (15.38***)**	**.05 (13.76***)**	**.06 (15,76***)**	**.05 (14.73***)**
CE	**.08 (.30***)**	**.17 (55.56***)**	**.04 (10.91***)**	**.09 (27.21***)**	**.06 (18.03***)**	**.17 (54.43***)**	**.01 (3.72*)**	**.06 (15.20***)**

Notes. Significant effects are highlighted in bold. GERM = German as main-domestic language; SES = socioeconomic status; PL = phonological loop; CE = central executive; $n = 303$; *= $p \leq .05$, ** = $p \leq .01$ *** = $p \leq .001$; $n = 303$.

Supplementary Table 9. *Univariate ANCOVAs of linguistic competencies with direct vs. indirect measurement of central executive*

	central executive (direct)	central executive (indirect)	central executive (direct)	central executive (indirect)	central executive (direct)	central executive (indirect)
Vocabulary	t1		t2		t3	
	η_p^2 *(F)*	η_p^2 *(F)*	η_p^2 *(F)*	η_p^2 *(F)*	η_p^2 *(F)*	η_p^2 *(F)*
Model	**.60 (50.93***)**	**.62 (56.03***)**	**.42 (24.85***)**	**.43 (25.91***)**	**.30 (14.32***)**	**.30 (14.19***)**
Group	**.41 (63.05***)**	**.43 (68.88***)**	**.25 (30.78***)**	**.28 (35.98***)**	**.11 (10.81***)**	**.14 (13.82***)**
GERM	**.08 (24.60***)**	**.10 (29.27***)**	.00 (.45)	.00 (.50)	**.02 (6.32*)**	**.02 (6.18*)**
SES	.00 (1.07)	.01 (1.60)	**.04 (11.55***)**	**.04 (12.03***)**	**.01 (2.75^{+})**	**.02 (3.98*)**
Sex	**.04 (10.35**)**	**.04 (11.02***)**	.01 (2.62)	.01 (3.47)	.01 (2.73)	.01 (3.11)
PL	.01 (2.56)	.01 (2.41)	.01 (1.81)	.01 (2.42)	**.01 (3.0^{+})**	**.02 (4.71*)**
CE	.00 (.79)	.01 (2.41)	**.03 (7.34**)**	**.02 (6.51*)**	***.03 (7.93*)***	.01 (2.73)
Grammar	t1		t2			
	η_p^2 *(F)*	η_p^2 *(F)*	η_p^2 *(F)*	η_p^2 *(F)*		
Model	**.55 (42.12***)**	**.54 (40.88***)**	**.44 (26.33***)**	**.43 (26.11***)**		
Group	**.40 (61.48***)**	**.42 (67.43***)**	**.15 (16.50***)**	**.20 (21.45***)**		
GERM	.00 (.32)	.00 (.63)	.00 (.02)	.00 (.00)		
SES	**.01 (3.74^{+})**	**.02 (6.14*)**	**.04 (12.62***)**	**.05 (15.22***)**		
Sex	.00 (.90)	.00 (1.14)	.01 (2.17)	.01 (2.16)		
PL	**.03 (7.20**)**	**.04 (10.77***)**	**.10 (30.24***)**	**.12 (36.73***)**		
CE	**.06 (16.05***)**	**.02 (4.41*)**	***.04 (11.30***)***	.01 (2.50)		

Notes. Significant effects are highlighted in bold. Cursive effects are divergent effects of the initial central executive measurement, which was not used in our analyses due to the unacceptable reliability ($\alpha < .50$). The initial central executive measurement was collected verbally (digit

backward), the utilized central executive measurement was collected nonverbally. GERM = German as main domestic language; SES = socioeconomic status; PL = phonological loop; CE = central executive; n = 303; *= $p \leq .05$, ** = $p \leq .01$, *** = $p \leq .001$.
